# GRIEF EXPERIENCE PATTERNS AMONG OLDER ADULTS IN RURAL CHINA: A LATENT PROFILE ANALYSIS

**DOI:** 10.1093/geroni/igz038.2347

**Published:** 2019-11-08

**Authors:** Haimin Pan

**Affiliations:** Xiamen University, Xiamen, China, China

## Abstract

Grief experiences among older adults in China are understudied, though a variety of negative bereavement outcomes have been delineated. The present work sought to explore grief patterns among Chinese older people in rural areas, as well as the factors influencing the bereavement results. Participants were 352 older residents who responded to a face-to-face interview and lived in rural areas in Zhejiang Province of China. Latent profile analysis (LPA) was used to identify subtypes of class membership in combing complicated grief (CG), depression, anxiety, and meaning in life. Afterwards, these subgroups were compared on demographic characteristics and meaning making variable. The LPA model best fitting the data was a three-class solution comprised of “adaptive” (n=235; 66.8% of the sample), “moderate maladaptive” (n=83; 23.6% of the sample), and “severe maladaptive” groups (n=34; 9.7% of the sample). Compared to the “severe maladaptive” group, participants in the “adaptive” group had better physical functioning, higher education and incomes levels, and less meaning making engagement, while participants in the “moderate maladaptive” group had longer bereavement duration, better physical functioning, and less meaning making activities. Relative to the “moderate maladaptive” group, participants who were adaptive to the loss possessed longer bereavement duration better physical functioning, higher education and incomes levels, and less meaning making engagement. Findings suggest three distinct patterns of bereavement outcomes among Chinese older adults. Multiple factors impacting the results were taken into consideration. Future replication is necessary to validate these subgroups, and professional services should be provided to bereaved older Chinese in need.

